# Lévy statistics define anxiety and depression in mice subjected to chronic stress

**DOI:** 10.3389/fnbeh.2026.1757347

**Published:** 2026-07-21

**Authors:** Qinxi Li, Xiaojie Li, Qixing Zhong, Yuru Nie, Yiping Luo, Bangcheng Zhao, Ni Zhang, Weihong Kuang, Chao Tian, Daojun Chen, Yingqian Zhang, Zhe Wu, Zhihui Zhong

**Affiliations:** 1 Laboratory of Neurological Disease Modeling and Translational Research, West China Hospital, Sichuan University, Chengdu, China; 2 Department of Neurology, West China Hospital, Sichuan University, Chengdu, China; 3 Tianfu Jincheng Laboratory, City of Future Medicine, Chengdu, Sichuan, China; 4 School of Life Sciences and Technology, University of Electronic Science and Technology of China, Chengdu, Sichuan, China; 5 Sichuan Provincial Center for Mental Health, The Center of Psychosomatic Medicine of Sichuan Provincial People’s Hospital, University of Electronic Science and Technology of China, Chengdu, China; 6 Sichuan Junhui Biotechnology Co., Ltd., Chengdu, China; 7 Department of Psychiatry, West China Hospital, National Chengdu Center for Safety Evaluation of Drugs, Sichuan University, Chengdu, China; 8 Department of Psychiatry, West China Hospital of Sichuan University, Chengdu, Sichuan, China; 9 Department of Women’s Health, Sichuan Cancer Hospital, Sichuan, China; 10 Sichuan Provincial Center for Mental Health, Sichuan Provincial People’s Hospital, School of Medicine, University of Electronic Science and Technology of China, Chengdu, China

**Keywords:** anxiety, behavior, depression, Lévy flight, stress

## Abstract

**Introduction:**

Current rodent models for anxiety and depression assessment face methodological challenges compromising both scientific rigor and animal welfare.

**Methods:**

This study introduced a novel approach using the Lévy flight (LF) statistical method to analyze spontaneous movement in open spaces. We employed three models: Chronic unpredictable mild stress (CUMS), Electric shock stress (ES), and Chronic Restraint Stress (CRS)—utilizing a total of 540 mice for LF fitting. A support vector machine algorithm was applied to distinguish each model group based on the two-dimensional distribution of the variables γ and μ in the LF. Statistical analysis was performed using a two-dimensional Kolmogorov-Smirnov test before and after drug administration.

**Results:**

We found that the ES model primarily exhibited anxiety-like behaviors, the CRS model predominantly exhibited depression-like behaviors, and the CUMS model displayed both depression-like and anxiety-like behaviors. All three stress models were suitable for LF fitting, with the distribution of CUMS in the γ-μ plane lying between the ES and CRS groups. To assess the therapeutic effect of fluoxetine (FXT) on CUMS, we excluded the CUMS-resistant mice and performed LF analysis. Following FXT treatment, the mice gradually shifted toward the normal area in the γ-μ plane, with a more pronounced shift toward the depression area observed as the modeling time increased.

**Discussion:**

This study identifies a previously unrecognized statistical locomotor pattern in mice with anxiety and depression. By integrating scientific rigor with ethical considerations, this approach also presents a humane paradigm shift in preclinical assessment, accelerating translational breakthroughs in neuroscience research.

## 1 Introduction

In 2019, the World Health Organization reported that one out of every eight individuals worldwide lives with one or more mental disorders (Cieza et al., 2021). The COVID-19 pandemic resulted in a significant increase in depression and anxiety cases within a year (Zhou et al., 2019). Chronic stress is the principal environmental factor that triggers a decline in mood, leading to depression and anxiety (Yu et al., 2022).

Rodent models for stress responses have been extensively used since the early 1980s to simulate these conditions, including the Chronic Unpredictable Mild Stress (CUMS), Electric Shock Stress (ES), and Chronic Restraint Stress (CRS) models (Willner, 2017; Yan et al., 2024, Tezanos and Trejo, 2025). In CUMS and its derived models, mice or rats are subjected to constant but unpredictable mild stressors, resulting in the development of depression-like or anxiety-like behaviors, mimicking the core symptoms of clinical depression and anxiety, such as anhedonia and acquired helplessness (Nestler and Hyman, 2010). CUMS has demonstrated its excellent reliability and validity in the realm of drug discovery (Bondi et al., 2008). However, the biological mechanisms underlying depression and anxiety disorders remain poorly understood, and there are no specific biomarkers. As a result, assessment primarily relies on behavioral studies in rodent models.

Rodents’ response to stress is typically observed through sequential behaviors, including anhedonia, abnormal weight, and poor coat condition. In order to quantitatively assess the severity of mental disorders and distinguish between anxiety and depression-like behaviors in experimental animals, conventional neurobehavioral studies such as the open field test (OFT), elevated plus maze (EPM) and light/dark box (LDB) test for anxiety (Bondi et al., 2008; Alves et al., 2018). The sucrose preference (SP) test, forced swim test (FST) and tail suspension test (TST) for depression are commonly employed (Kim and Han, 2006; Cryan et al., 2005). Nevertheless, it is important to acknowledge that each of these established methodologies has its own set of limitations. For instance, Cheryl D. Conrad has pointed out that the assessment of anxiety through the OFT does not necessarily correlate with anxiety assessed using the EPM (Du Preez et al., 2021). Additionally, the TST and FST assays only capture a single facet of depression (Cryan et al., 2005), while the EPM may induce anxiety in animals during testing, thereby complicating the evaluation process (Tomida et al., 2009). Furthermore, the very procedures involved in these experiments impose additional stress on the animals (Kraeuter et al., 2019), which can interfere with the interpretation of data and restrict the translational relevance of the studies. The rise of the artificial intelligence era has significantly increased the popularity of behavioral research, particularly in the analysis of rodent behavior using mathematical models and artificial intelligence algorithms. For example, in 2021, Professor Wang Liping’s team proposed the use of 3D behavior analysis to examine the detailed activities of rodents (Huang et al., 2021). Weinreb et al. (2024) integrated point tracking with posture dynamics through mathematical fitting to analyze rodent behavior. These deep learning methods represent a promising direction for future development; however, they currently face challenges such as high computational costs and limited throughput. We sought to distinguish between depressed and anxious mice in a simple and efficient manner, enabling high-throughput drug screening.

Lévy flight (LF), named after the esteemed French mathematician Paul Levy, is a “random walk” initially denoted a stochastic traversal characterized by a heavy-tailed probability distribution of step lengths. Within mathematics, the “random walk” is an algorithm that takes cues from nature, involving a sequential progression of successive steps with random distances or velocities (Barthelemy et al., 2008; Dannemann et al., 2018). In the natural world, the movement of animals exhibits different patterns based on the availability of food. When resources are plentiful, animal trajectories tend to resemble “Brownian motion,” characterized by frequent revisits to previously explored areas, a phenomenon often termed “oversampling” (Viswanathan, 2010). In contrast, animals opt for more extensive movements when food and resources become scarce, manifesting as “heavy tails” in the probability distribution of step lengths. In statistical terms, “heavy tails” describes the phenomenon wherein the tails of a probability distribution exhibit slower decay. This implies that extreme events or outliers are more likely to occur than distributions with lighter tails (Lindquist and Rachev, 2021). The “LF foraging hypothesis” suggests that this motion pattern enhances their chances of survival (Dannemann et al., 2018). The applications of LF encompass a broad spectrum, including the analysis of earthquake data, financial mathematics, cryptography, signal analysis, and a wide array of practical applications in the fields of astronomy, biology, and physics (Huda et al., 2018; Raichlen et al., 2014).

The hypothesis in this study posits that laboratory animals would transition from “Brownian” to “LF-like” motion patterns due to evolutionary pressure encoded in their genetic makeup. In this study, our objective is to examine the potential of “LF-like” motion patterns in distinguishing between stressed and non-stressed mice and further evaluate their effectiveness in differentiating anxiety and depression mice within the stressed group. These methodologies possess practical implications in the development of a streamlined single-step experimental technique for quantifying the severity of anxiety and depression induced by stress. Moreover, this approach holds the potential to minimize the extra stress encountered by animals during repetitive experimental protocols while concurrently enhancing the efficacy of drug discovery endeavors.

## 2 Materials and methods

### 2.1 Animals

Experiments were conducted using 685 male and female C57Bl/6 mice (6–8 weeks old, weighing 18–24 g) obtained from Charles River Laboratories Animal Technology Co., Ltd. (Beijing, China). The animals were housed under specific pathogen-free conditions, provided standard laboratory chow and distilled water, and maintained on a 12-h light-dark cycle in standard cages with corn cobs in a room maintained at an ambient temperature of 23 ± 1°C at Junhui Biotech, Co., Ltd. (Sichuan, China) facilities [animal production license number: SYXK (Chuan) 2019-215]. All procedures followed the guidelines of the Association for Assessment and Accreditation of Laboratory Animal Care (AAALAC) and were approved by the Institutional Animal Care and Use Committee (IACUC) of the West China Hospital, Sichuan University (Approval No. 2019195A).

### 2.2 Chronic unpredictable mild stress (CUMS)

The CUMS modeling process was adjusted slightly. It consisted of sequential application of mild stressors, including cage tilting, heat stress, noise stimulation, wet bedding, loneliness, fasting, inversion of a day-night cycle for 24 h, water deprivation, reciprocating sway, random foot shock for 120 s, crowding, cold stress, flash stimulation for 12 h, constraint for 4 h, and cage replacement for 24 h. Each stimulus was randomly arranged, and two different stressors were performed consecutively daily throughout the experiment (Bondi et al., 2008). The model is maintained until the completion of the behavioral experiment.

### 2.3 Chronic restraint stress (CRS)

The CRS protocol underwent slight modifications compared to previous descriptions (Pfeiffer, 1967, Seo et al., 2017). The establishment of a mouse model of depression often involves the common utilization of CRS. One week before the start of stress exposure, animals were divided into control and experimental groups to acclimate to new cages. Mice were placed in a cylinder for 4 h daily (8:00–12:00) for 28 consecutive days, almost immobilizing them. The size of the cylinder matched the size of the animal. Non-stressed controls were moved to a test room and handled gently for 5 min before returning to their holding room 4 h later.

### 2.4 Electric shocks (ES)

The ES modeling session was executed following prior descriptions, incorporating minor modifications. Electric foot shocks were employed to induce an anxiety mouse model (Zhang et al., 2020). After a 30-min adaptation, mice underwent ten intermittent inescapable electric foot shocks delivered by an isolated shock generator (Shanghai Jilang Information Technology Co., Ltd., China) through the grid floor. The shocks had an intensity of 0.8 mA, an interval of 15 s, and a duration of 15 s. Control mice were placed in the same chamber for 5 min without undergoing electric foot shock (Zhang et al., 2020).

### 2.5 Grouping

This study comprised two sets of experiments. In the first set, we established three stress models—CUMS, ES, and CRS—incorporating a total of 540 mice, with 237 in the control group, 145 in the CUMS group, 89 in the ES group, and 69 in the CRS group. In the second set, we screened for stress-resistant mice within the CUMS model. The specific procedure is as follows:

Before CUMS induction, all mice underwent a SP test and an OFT to preclude physically and mentally low activity subjects. Before the experiment (day-2), we evaluated the sucrose preference of mice. Based on the preference rate, we excluded 11 discrete mice. At the onset of the modeling process, the CUMS modeling group was assigned the designation D0. On Day 0, the remaining 134 mice underwent an OFT, excluding 31 that made fewer than five entries into the center zone. Subsequently, a total of 103 mice were allocated into two groups, namely the control group comprising 32 mice and the CUMS modeling group consisting of 71 mice (as the CUMS modeling group will later be divided into CUMS + Saline group and CUMS + Fluoxetine (FXT) group). On D14, we conducted model validation by performing additional tests on SP and the OFT. Control mice with a sucrose preference percentage lower than 80% and CUMS mice with a ratio higher than 80% were excluded from the SP test. In the OFT, CUMS mice that crossed the center zone more than five times were also excluded. Unfortunately, throughout the experiment, we experienced two deaths in the control group and four deaths in the CUMS group. Consequently, we had a total of 27 remaining mice in the control group and 52 successfully modeled mice in the CUMS group. On D14, as part of the model validation process, we performed additional SP and OFT experiments. Control mice with a sucrose preference percentage below 80% and CUMS mice with a sucrose preference percentage above 80% were excluded from the SP test. In the OFT, CUMS mice that crossed the center zone more than five times were excluded. Regrettably, during the experiment, two deaths occurred in the control group, and four deaths occurred in the CUMS group. Consequently, there were a total of 27 mice remaining in the control group and 52 successfully modeled mice in the CUMS group. Following Day 14, we partitioned the CUMS group into CUMS + Saline and CUMS + FXT groups. The CUMS + Saline group was administered 5 mL/kg of Saline each day after modeling. In contrast, the CUMS + FXT group was given a daily dosage of 20 mg/kg of Fluoxetine hydrochloride after modeling. Treatment was sustained until the culmination of the experiment (Supplementary Figure 1).

### 2.6 Physical health assessment

Body weight, food intake, and coat state score were evaluated weekly, specifically on Wednesdays between 10 a.m. and 12 a.m., until the conclusion of day 49. The 24-h food consumption was meticulously documented. The assessment of the coat state score encompassed seven distinct areas, namely the head, neck, dorsal region, ventral coat, tail, anterior claw, and hind claw. This evaluation aimed to identify any signs of deterioration in the physical condition of the coat, such as fur loss and accumulation of dirt (Alonso et al., 2004).

### 2.7 Sucrose preference (SP) test

In preparation for the SP test, 1% sucrose solution and distilled water were placed simultaneously in each mouse’s cage 2 days before the test for taste adaptation. On the third day, following a 6-h fast and water deprivation, mice were raised individually in cages with one pre-weighed bottle of 1% sucrose solution and another of distilled water. After 6 h, the positions of the two bottles were interchanged. Subsequently, after an extra 12 h, both bottles were eliminated, and the residual liquid was quantified to ascertain mouse sucrose preference, employing the subsequent Equation 1 (Liu et al., 2018).


S⁢u⁢c⁢r⁢o⁢s⁢e⁢p⁢r⁢e⁢f⁢e⁢r⁢e⁢n⁢c⁢e⁢p⁢e⁢r⁢c⁢e⁢n⁢t⁢a⁢g⁢e= (1)

$$\;\,\frac{S\,u\,c\,r\,o\,s\,e\,c\,o\,n\,s\,u\,m\,p\,t\,i\,o\,n}{T\,o\,t\,a\,l\,l\,i\,q\,u\,i\,d\,c\,o\,n\,s\,u\,m\,p\,t\,i\,o\,n}\,\text{×}\,100\%$$

### 2.8 Behavioral assessment

The mice were acclimated to the testing room for 12 h before assessment. Maintaining a clean, odor-free, and quiet environment was crucial for the experiment, and the operator remained concealed during testing. The Topscan Package (Clever Sys Inc., United States) was used to record and analyze mouse movements.

#### 2.8.1 Open field test (OFT)

Each mouse was gently placed in a 50 * 50 * 50 cm OFT box for 5 min to explore freely under weak (50 lx) illumination. The total distance traveled (cm), and number of entries into the central area (25 * 25 cm) were recorded and analyzed using the software (Besnard et al., 2019).

#### 2.8.2 Elevated plus-maze test (EPM)

The EPM had two open and two closed arms. Each arm measured 30 cm in length and 5 cm in width, with walls that were 20 cm high in the closed arms. The EPM was elevated to a height of 60 cm above the ground, with a 90°angle between the arms. Prior to the test, mice were placed in the central area facing the open arm and allowed to explore for 5 min. Open Arm Entries Percent (OE%) was calculated as Entries into the Open Arm divided by (Entries into the Open Arm + Entries into the Closed Arm) multiplied by 100. Open Arm Time Percent (OT%) was calculated as time in the Open Arm divided by 300 s multiplied by 100 (Snyder et al., 2011).

#### 2.8.3 Light/dark box (LDB) test

Anxiety was tested in the LDB as previously described. Each mouse was placed in an apparatus consisting of two identical opaque Plexiglas compartments (18 * 12 * 12 cm) and an opening (5 cm * 5 cm). The mice were positioned in the light box with their heads facing the dark box, and their exploratory behavior was monitored for 10 min following their initial crossing (Zheng et al., 2009).

#### 2.8.4 Tail suspension test (TST)

The mice were suspended by their tails using adhesive tape hooked onto a horizontal rod. The distance between the tip of the mouse’s nose and the floor was approximately 25 cm. After being suspended for 6 min, the time spent immobile during the last 4 min was recorded (Tomida et al., 2009).

#### 2.8.5 Forced swimming test (FST)

Before testing, the mice were placed in plastic cylinders filled with water (25 ± 1°C; depth: 15 cm) for 15 min. The next day, the mice were placed in a 10-cm-wide cylinder (height: 30 cm), and the time spent immobile during the final 4 min of the 6-min forced swimming test was recorded (Yang et al., 2018).

### 2.9 LF fitting and Lévy distribution

The mice were placed in a standard OFT apparatus for behavioral studies (Clever Sys., Inc. United States), and their behavior was recorded through a series of videos. The recorded videos were analyzed using a custom MATLAB script (Math Works Inc., United States, version R2019a). The mice were segmented from the videos frame-by-frame, and the mean coordinates of the segmented pixels were found to determine the center of each mouse. Walking speed was calculated by measuring the distance a mouse traveled between a specific number of frames, divided by the time interval. For instance, a 5-min video recording of mouse behavior was provided. The video had a frame rate of 25 Hz, with 25 independent frames presented per second. The resolution of the video was 1,280 × 720 pixels, corresponding to high-definition (HD) quality. Resolution indicates the clarity and detail of the video, with 1,280 pixels along the horizontal axis and 720 pixels along the vertical axis. The higher resolution facilitates accurate tracking of the mice’s movements and outlines, offering high-quality visual data for subsequent analysis and processing. To maintain balance between measurement accuracy and sensitivity to speed changes, speed was calculated every five frames; we fitted two probability density functions (PDF) for the walking speed statistics of each mouse from all the categories. The first PDF is the LF distribution. A simplified and often employed form of the LF distribution is defined as Yang (2010):


$$L\,\left(s,\gamma ,\mu \right)=\left\{ \begin{array}{c} \sqrt{\frac{\gamma }{2\,\pi }}\,e^{\left[-\frac{\gamma }{2\,\left(s-\mu \right)}\right]}\,\frac{1}{{\left(s-\mu \right)}^{3/2}},0<\mu <s<\infty \end{array}\right.$$ (2)

L is the probability density, μ > 0 is the minimum possible speed, and γ is a scale parameter.

We perform a parametric curve fitting of the distribution data to Equation 2. This method enables us to convert an animal’s random walk into a pair of parameters; hence, it can be plotted as a dot in a two-parameter γ-μ plane.

The other PDF is the normal distribution (Yang, 2010):


$$f\,\left(s,\sigma ,\mu \right)=\frac{1}{\sqrt{2\,\pi }}\,e^{-\frac{{\left(s-\mu \right)}^{2}}{2\,\sigma^{2}}}$$ (3)

Statistical distributions can exhibit diverse shapes, yet they frequently adhere to typical patterns. Among these patterns, the normal distribution is particularly prevalent, characterized by a majority of values clustering around the mean and fewer values as they deviate from it, often referred to as the “tail” of the distribution. Both models depict random walks with distinct probability distributions in the context of LF and the normal distribution. Notably, the LF model possesses a distinctive feature whereby its distribution values in the far right region are slightly larger than those of the normal distribution.

The Goodness of Fit (R-good) is a quantitative representation of the curve fitting quality, which is defined as:


$$\text{R-good}=1-\frac{S\,S\,E}{S\,S\,T}$$ (4)

Where SSE (error sum of squares) is defined as SSE = $\sum_{i=1}^{n}{\left(x_{i}-\hat{x_{i}}\right)}^{2}$ and SST is defined as SST$=\sum_{i=1}^{n}{\left(x_{i}-\bar{x_{i}}\right)}^{2}.x_{i}$ is defined as the average value of this set of data. The value of R-good is between 0 and 1. The closer it is to one, the better the fit is.

### 2.10 Support vector machine (SVM)

The SVM is an algorithm used for classification. Its main goal is to find the best possible boundary that separates different clusters of data points in a way that maximizes the margin between them. It is suitable for the feature reduction of high-dimensional data with a small sample size. A general mathematical model of support vector machines is shown in Equations 5 and 6.


$$\min_{\omega ,\varepsilon }\frac{1}{2}\,\omega_{2}^{2}+C\,\sum_{i=1}^{n}\varepsilon_{i}\,l_{\text{i}}$$ (5)

$$s.t.\left(\omega^{T}\,\varphi \,\left(x_{i}\right)+b\right)\geq 1-\varepsilon_{i},\varepsilon_{i}\geq 0,n=1,\ldots ,n$$ (6)

The detailed solution process of the SVM problem is found in Noble (2006).

### 2.11 Fluoxetine (FXT)

Fluoxetine hydrochloride (Sigma Chemical Company, St. Louis, MO, United States) was dissolved in Saline and administered once daily via the intraperitoneal route for 37 days at 20 mg/kg. The fluoxetine hydrochloride dosage was selected based on previous studies (Pawluski et al., 2014).

### 2.12 Statistical information

The statistical analysis was done using IBM SPSS Statistics version 26.0 software (IBM Ltd., United Kingdom). Data that followed a normal distribution were presented as mean ± standard error of the mean (SEM). Unpaired *t*-tests were employed to compare the differences between the control and model groups. When the data followed a normal distribution and had equal variances, a one-way Analysis of Variance (ANOVA) followed by *post-hoc* Tukey Dunnett’s multiple comparison tests was used to analyze differences among the model group, buspirone group, and each treatment group. A repeated measures ANOVA was conducted to examine variations in body weight, food intake, coat state, sucrose preference, and fecal amount among groups. The interaction effects between repeated indicators and days were assessed using Pillai’s trace. If interactions were observed between a specific variable and days, the differences among each group were compared at the final time point. If no interactions were present, *post-hoc* analysis using Bonferroni’s multiple comparison tests was performed. The correlations between each effect were evaluated using Pearson’s correlation. Statistical significance was set at a two-sided *p*-value < 0.05.

The 2-Dimensional Kolmogorov–Smirnov (2D K-S) test is a nonparametric, bivariate statistical test designed to verify whether two two-dimensional samples follow the same distribution (Feyen et al., 2021). The point defined four quadrants for any given point in a two-dimensional space. The integration probability is defined as the probability of sample data distribution in one of the quadrants that accounted for the total number of samples. By iterating over all data points and quadrants, the test statistic D_*FF,1*_ is defined by the maximal difference of the integrated probabilities between samples in any quadrant for any origin from the first sample. Similarly, after going through all points of another sample, the test statistic D_*FF,2*_ is obtained. D_*FF,1*_ and D_*FF,2*_ are then averaged to compute the overall D_*FF*_ for hypothesis testing, D_*FF*_ = (D_*FF,1*_+D_*FF,2*_)/2.

In the large sample limit (*n* ≥ 80), it was shown that D_*FF*_ converged in distribution (Feyen et al., 2021).


$$D_{F\,F}\overset{d}{⟶}\Phi \,\left(\lambda \right)=2\,\sum_{k=1}^{\infty }{\left(-1\right)}^{k-1}\,e^{-2\,k^{2}\,\lambda^{2}}$$ (7)

For a single sample of size n


$$P\left(d_{F\,F}>D_{F\,F}\right)=\Phi \left(\frac{D_{F\,F}\,\sqrt{n}}{1+\sqrt{1-r^{2}}\,\left(0.25-0.75/\sqrt{n}\right)}\right)$$ (8)

The two-sample case used the same formula as above, where n is defined as


$$n=\frac{n_{1}\,n_{2}}{n_{1}+n_{2}}$$ (9)

and r is defined as


$$r=\frac{\sum_{i}\left(X_{i}-\bar{X}\right)\,\left(Y_{i}-\bar{Y}\right)}{\sqrt{\sum_{i}{\left(X_{i}-\bar{X}\right)}^{2}}\,\sqrt{\sum_{i}{\left(Y_{i}-\bar{Y}\right)}^{2}}}$$ (10)

## 3 Results

### 3.1 Establishment of CUMS, ES, and CRS models for evaluating depression and anxiety in mice

**FIGURE 1:**
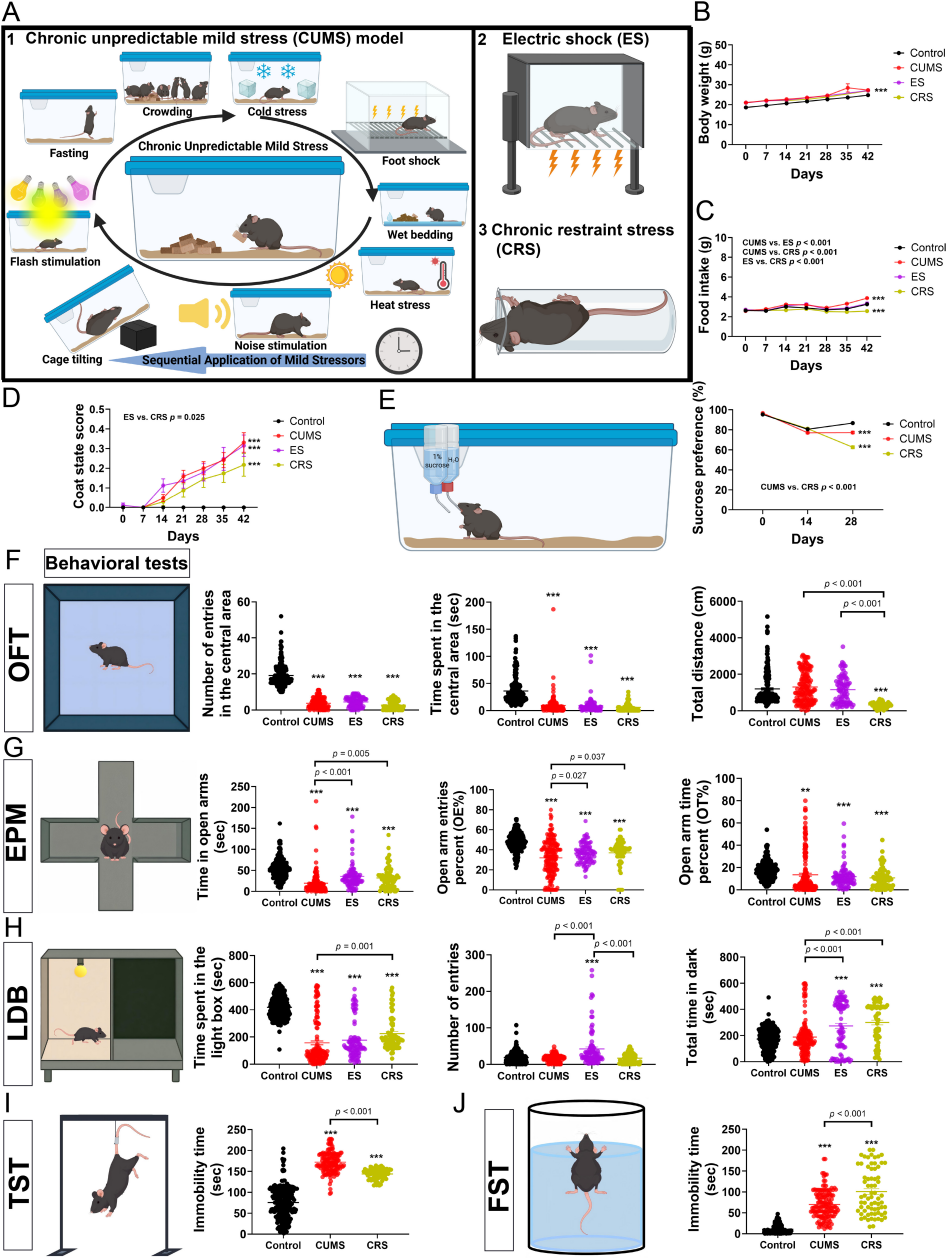
Establishment of CUMS, ES, and CRS models for evaluating depression and anxiety in mice. **(A)** Schematic representation of the three models: 1. Chronic unpredictable mild stress (CUMS), 2. Electric shocks (ES), 3. Chronic restraint stress (CRS). **(B**–**D)** Body weight, food intake, and coat state score of mice in each group throughout the modeling and behavioral testing period. **(E)** Left panel: Schematic illustration of the sucrose preference test protocol. Right panel: Quantification of sucrose preference in mice exposed to CUMS or CRS paradigms. **(F**–**J)** Behavior of the three groups of model mice across the open field test (OFT), elevated plus maze (EPM), light/dark box (LDB), tail suspension test (TST), and forced swim test (FST) indicators. Data are presented as mean ± SEM, with group sizes as follows: Control = 237, CUMS = 145, ES = 89, CRS = 69. One-way analysis of variance (ANOVA) was conducted, followed by Tukey’s *post-hoc* test for multiple comparisons. Statistical significance is indicated as: *p* < 0.05, **p < 0.001, and ***p < 0.0001 versus the control group.

Three widely used stress mouse models were established: CUMS and CRS as classic methods for modeling depression, and ES as a commonly utilized anxiety model (Figure 1A). During the modeling process and behavioral testing, the weight, food intake, and coat state scores of each group of mice were individually recorded. Compared to the control group, the body weight, food intake of the CUMS group was significantly increased (*p* < 0.001, Figures 1B,C). In contrast, the CRS group exhibited significantly lower food intake compared to the control group (*p* < 0.001, Figure 1C). For coat state scores, all three model groups had higher scores compared to the control group, with the ES group showing significantly higher scores than the CRS group (*p* = 0.025, Figure 1D). We also performed the sucrose preference test in the CUMS and CRS groups. Both models showed significant anhedonia relative to controls, and sucrose preference was significantly lower in the CRS group than in the CUMS group (*p* < 0.001, Equation 1 and Figure 1E). In the OFT, the CUMS, ES, and CRS groups showed significantly higher values in the “number of entries into the central area” and “time spent in the central area” compared to the control group (*p* < 0.001). However, the CRS group covered a shorter total walking distance than the other three groups (*p* < 0.001, Figure 1F). In the EPM test, significant differences were observed in the “Time in open arms” index between CUMS and the ES and CRS groups (ES: *p* < 0.001; CRS: *p* = 0.005). In the OE% indicator, significant differences were observed between CUMS and both the ES and CRS groups (*p* = 0.027 and *p* = 0.037, respectively, Figure 1G). In the LDB test, compared to the control group, the time spent in the bright box was significantly reduced in the CUMS, ES, and CRS groups (Figure 1H, left). Interestingly, the crossing frequency of ES mice was significantly higher than that of the other three groups (Figure 1H, middle). Additionally, the time spent in the dark box was significantly longer for the CUMS group compared to the other groups (Figure 1H, right). In the TST, both the CUMS and CRS groups showed increased immobility time, with the CUMS group exhibiting significantly higher immobility than the CRS group (*p* < 0.001, respectively, Figure 1I). Interestingly, in the FST, the CRS group displayed significantly higher immobility time than the CUMS group (*p* < 0.001, respectively, Figure 1J). Our results demonstrate that the CUMS and CRS models exhibited anhedonia and despair behaviors, with significant abnormalities across various indicators in the OFT, EPM, and LDB tests compared to the control group. The ES model, on the other hand, displayed anxiety-like behaviors in the OFT, EPM, and LDB tests. However, unfortunately, the existing behavioral criteria do not allow for accurate differentiation between isolated depressed or anxious mice.

### 3.2 The velocity of mice walking in an OFT showed a pattern of LF

**FIGURE 2:**
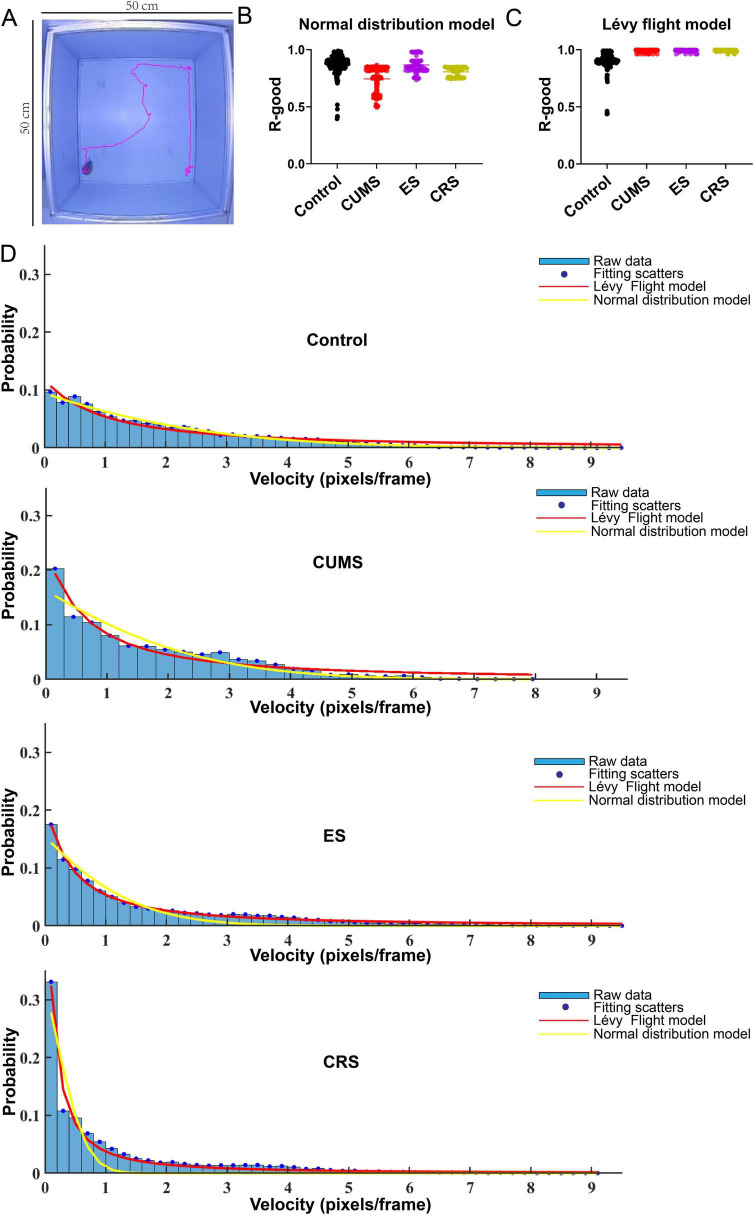
The velocity of mice walking in an OFT showed a pattern of LF. **(A)** Schematic representation of a mouse freely navigating a 50 × 50 cm open field arena. **(B,C)** The comparison between the r-good of the normal distribution **(B)** and LF distribution model **(C)** was performed in each group. **(D)** A control mouse, a CUMS mouse, an ES mouse, and a CRS mouse were randomly selected for application of the normal distribution and LF distribution models.

To address the challenge of distinguishing mice with depressive and anxious behaviors, we aim to utilize the OFT, a widely employed method in behavioral research, to assess the spontaneous locomotor activity of mice and thereby differentiate between depressive and anxious models (Figure 2A). The R-good quantitatively represented curve fitting quality. Results showed that control mice’s walking behavior followed normal and LF distributions, while CUMS, ES and CRS mice had an LF distribution, as depicted in Figures 2B,C and Equations 2–4. The frequency of walking speed was computed and represented as a bar graph in Figures 2B,C. Additionally, two probability density functions (PDFs), the LF distribution and normal distribution, were fitted to the walking speed statistics for each mouse category (Figure 2D). Therefore, we ultimately selected the LF model as the standardized evaluation method for all four groups.

### 3.3 The distribution of each group of model mice across the two variable parameters of the LF

**FIGURE 3:**
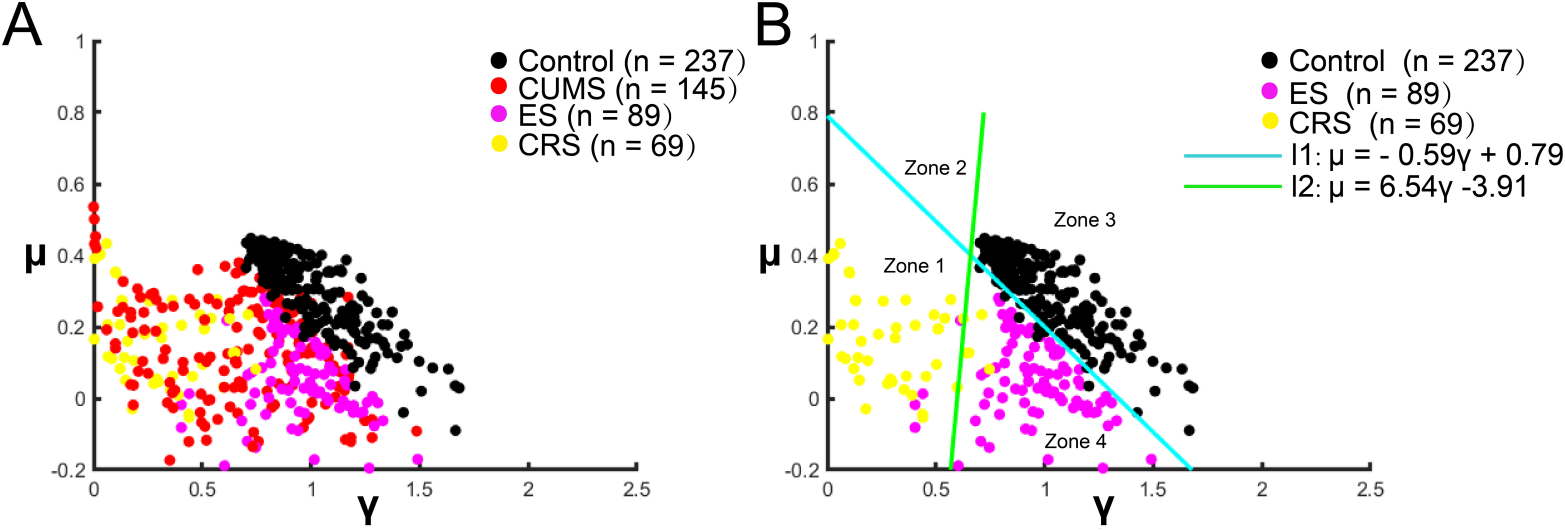
The distribution of each group of model mice across the two variable parameters of the LF. **(A)** The distribution of the two variable parameters of the LF (γ and μ). **(B)** A support vector machine was used to calculate two decision boundaries that distinguished the three data groups based on training data. The optimal linear separation between the control and CRS groups was defined by L1: μ = –0.59γ + 0.79, while L2: μ = 6.54γ –3.91 provided the optimal separation between the control and ES groups.

The LF mathematical model includes two key variable parameters, γ and μ, as outlined in Method (LF, Equation 2). After performing LF fitting for each mouse in the Control, CUMS, ES, and CRS groups, the γ and μ values were calculated. Each mouse was then represented as a point on the γ-μ plane (Figure 3A). We observed that in the γ-μ plane, the distributions of the Control, ES, and CRS groups were relatively concentrated, whereas the distribution of the CUMS group was more scattered, positioned between the ES and CRS groups. Therefore, we aimed to classify the data on the γ-μ plane into Control, ES, and CRS groups. To achieve this, we applied the SVM method (Equations 5, 6). Given the data’s distribution, we used the SVM algorithm to find the optimal separation of the three mice groups in the γ–μ planes. The optimal linear separation between the Control and CRS groups was defined by L1: μ = –0.59γ + 0.79, while L2: μ = 6.54γ–3.91 represented the optimal separation between the Control and ES groups (Figure 3B). The γ-μ planes of L1 and L2 were divided into four distinct zones (1, 2, 3, and 4). Our analysis revealed that CRS was predominantly concentrated in Zone 1, ES in Zone 2, and Control in Zone 3.

### 3.4 Mice exposed to CUMS displayed characteristic anxiety-like or depression-like behaviors

**FIGURE 4:**
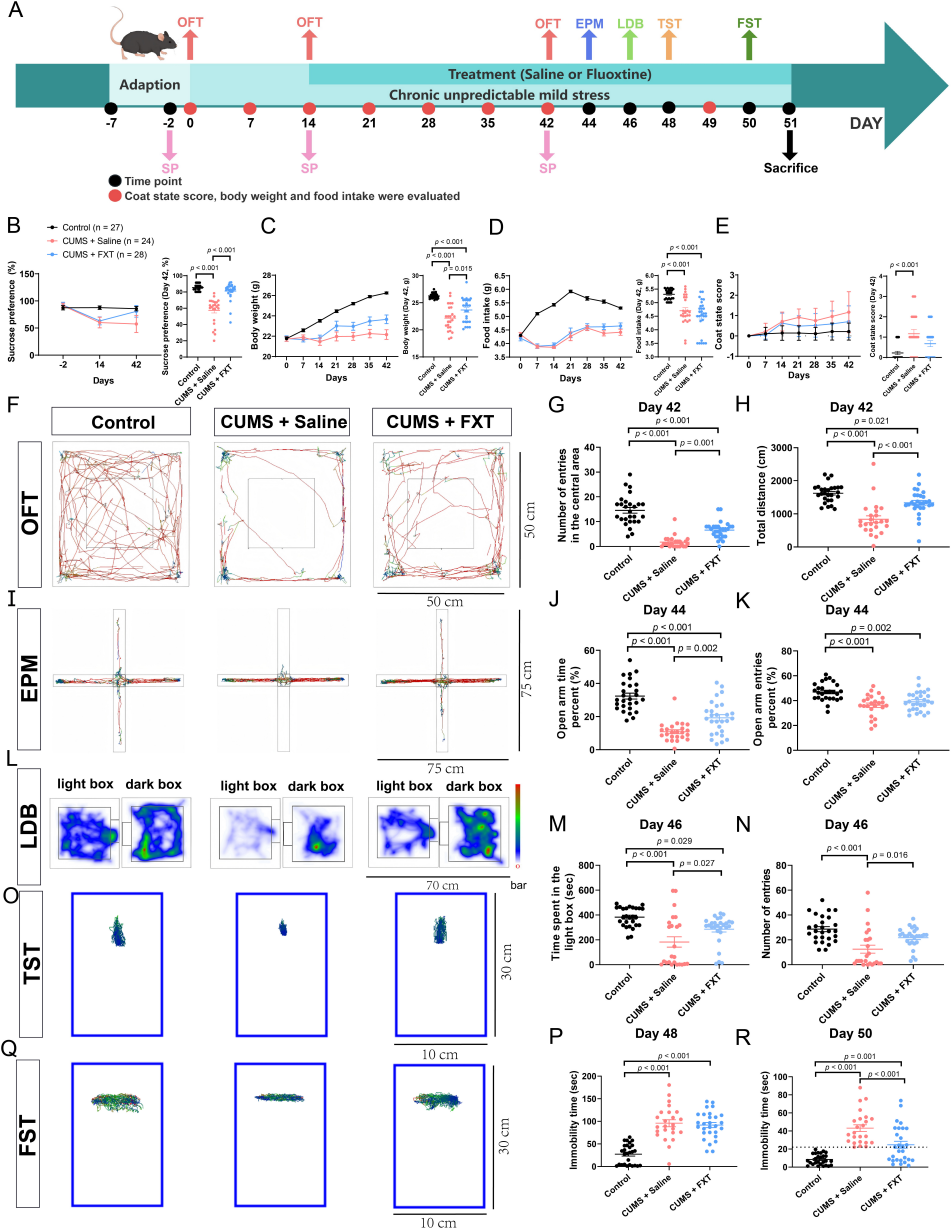
Timeline and validation of the experimental design for the CUMS Model. **(A)** Establishment of the CUMS mouse model, administration of the positive control drug fluoxetine, and behavioral testing period. **(B)** As depicted in the left Figure, exposure to the CUMS paradigm resulted in a substantial manifestation of anhedonia, as evidenced by the SP test. Additionally, on the 42nd day of the experiment, discernible differences in the percentage of water preference were observed among the various groups, as displayed in the right Figure. **(C**–**E)** In the left Figure, the CUMS paradigm was found to induce significant alterations in coat state score, body weight, and food intake. Furthermore, on the 42nd day of the experiment, the right Figure depicts a comparison of variations among the three groups. **(F)** On the 42nd day, OFT trajectories were recorded and analyzed. **(G)** On the 42nd day, mice in the CUMS + FXT group exhibited a higher number of center entries during the OFT. **(H)** CUMS + FXT mice exhibited reduced anxiety- and depression-like behaviors, as indicated by significantly greater distances traveled during the OFT **(I,J)**. During the EPM test, CUMS + Saline group spent less time exploring the open arm, with a notable difference observed between the CUMS + Saline and CUMS + FXT groups. **(K)** No significant difference in behavior was observed between the CUMS + Saline and CUMS + FXT groups. **(L,M)** CUMS + Saline group exhibited significantly reduced exploration time in the light box during the LDB test. **(N)** On the 46th day, the control group had a significantly greater entry during the LDB test. **(O–R)** During the TST and FST, mice in the CUMS + Saline group exhibited significantly more extended periods of immobility. The presented data represent mean ± S.E.M. Statistical significance was established when *p*-values were below 0.05. The number of animals is indicated on the graphs. A repeated measures ANOVA was employed to assess potential disparities among groups. In cases where interactions between an index and days were observed, we evaluated the distinctions among each group’s final time points, encompassing factors such as SP test outcomes, coat state scores, body weight, and food intake. In instances where no interactions were detected, subsequent *post-hoc* analysis, employing Bonferroni’s multiple comparison tests, was conducted.

We found that in the γ-μ plane of LF, the CUMS group is distributed between the ES and CRS groups, yet it can be distinguished from the Control group. We are considering conducting further analysis to explore the role of the CUMS model within the LF framework. We established the CUMS mouse model and administered the drugs to the groups on the 14th day post-model establishment. Anxiety behavior tests (OFT, EPM, and LDB) and depression behavior tests (SP, TST, and FST) were conducted at 6 weeks after model establishment. Additionally, body weight, food intake, and coat condition scores were measured weekly (Figure 4A). To mitigate the impact of CUMS-resilient mice on the experimental outcomes, we first used the SP test to exclude mice that did not show sensitivity to the SP test. Additionally, OFT were conducted prior to the modeling phase, both to verify the baseline for LF analysis and to exclude naturally inactive mice (Supplementary Figure 1A). Fourteen days after model establishment, we conducted the SP and OFT tests and excluded mice with unsuccessful model establishment by comparing their indicators to those of the control group (Supplementary Figures 1C,D). The SP test conducted on the 42nd day, the CUMS + FXT sucrose preference percentage exhibited a significant increase when compared to the CUMS + saline group (Figure 4B). The mice subjected to CUMS for 42 days showed lower body weight. In the CUMS + FXT group, the body weight on the 42nd day exhibited a significant increase compared to the CUMS + saline group (Figure 4C). On the 42nd day, the control group demonstrated a significantly elevated food intake in comparison to the CUMS + saline group (Figure 4D). In contrast, the control group exhibited a notably lower coat state score than that of the CUMS+saline group (Figure 4E). Nevertheless, no significant disparities were observed in the food intake and coat state score between the CUMS + saline group and the CUMS + FXT group. During 37 days of treatment, the success of the CUMS model was validated by administering FXT to CUMS mice, with the observation that the CUMS + Saline mice showed fewer center entries and distance during OFT (Figures 4F–H). Neurobehavioral tests, including EMP and LDB, were conducted on all mice from day 44 to day 50 to evaluate anxiety-like behavior. Additionally, TST and FST were employed to assess depressive-like behavior. The CUMS + Saline group exhibited less exploration time in the open arm of EPM and light box in LDB (Figures 4I–N), as well as reduced struggling time in TST and FST, as shown in Figures 4O–R. Conversely, FXT treatment increased the number of struggle times in the CUMS + FXT group. Based on the above results, we observed that the CUMS model induced depressive and anxiety-like behaviors, which were alleviated following treatment with FXT.

### 3.5 Validation of the CUMS model in the LF paradigm

**FIGURE 5:**
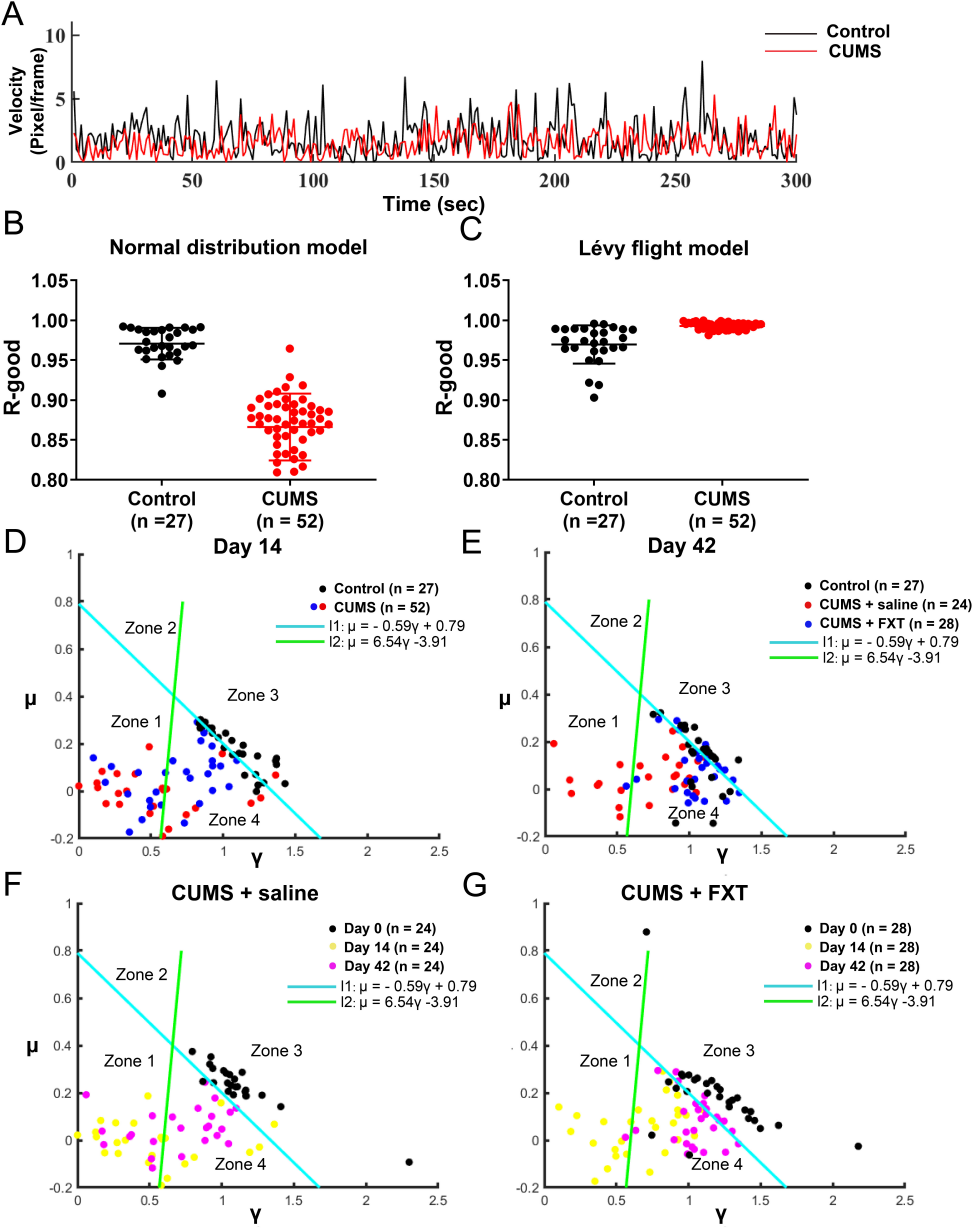
Validation of the CUMS model in the LF paradigm. **(A)** A mouse was selected randomly, and the walking speed of the two groups was recorded as a function of time. **(B,C)** Random selection was performed for a control mouse and a CUMS mouse to apply the control distribution and LF distribution models. **(D)** The black dots on the γ−−μ plane represented control mice (*n* = 27), while red and blue dots represented CUMS mice (*n* = 52). Our analysis indicated that LF patterns in the OFT distinguished Control and CUMS mice. The OFT data for the two groups were analyzed at the 14th-day time point after CUMS modeling. A substantial difference between control and CUMS mice was observed on the γ−−μ plane. **(E)** After day 14, CUMS-stressed mice were divided into CUMS + Saline and CUMS + FXT groups. On day 42, a significant difference between the CUMS + Saline and CUMS + FXT groups was observed. The comparison between Control and CUMS + Saline during OFT indicated a substantial difference, while no significant difference was found between Control and CUMS + FXT groups. **(F)** The OFT data for the CUMS + Saline group were compared using the same method at 0, 14, and 42 days. **(G)** Significant differences were observed in pairwise comparisons of OFT data at the three-time points in the CUMS + FXT group. The number of animals is indicated on the graphs. The null hypothesis that the 2D sets of variables followed the same distribution was tested using the two-dimensional Kolmogorov-Smirnov test.

We also applied the methods outlined in Section 3.2 to perform LF mathematical fitting on the validated CUMS model. In this study, the walking rates of each mouse group were plotted against time in Figure 5A. Results showed that control mice’s walking behavior followed normal and LF distributions, while CUMS mice had an LF distribution, as depicted in Figures 5B,C. Control mice were represented with black dots, while CUMS mice on day 14 were depicted with red and blue dots in Figure 3A. The 2D K-S test was utilized to determine whether the two-dimensional variables followed the same distribution (Equations 7–10). Results indicated a substantial difference between the control and CUMS groups (*p* < 0.001, Figure 5D). The CUMS-stressed mice were divided into CUMS + Saline and CUMS + FXT groups after day 14. On day 42, there was a significant difference between the CUMS + Saline and CUMS + FXT groups (*p* < 0.001, Figure 5E). Furthermore, compared to the control, the CUMS + Saline group exhibited more considerable differences during OFT (*p* < 0.001, Figure 5E). In contrast, no significant difference was observed between the control and CUMS + FXT group. Similar methods were used to compare OFT data of the CUMS + Saline group on day 0, 14, and 42, revealing significant differences between day 0 and day 14 (*p* < 0.001, Figure 5F), day 14 and day 42 (*p* = 0.040, Figure 5F), and day 0 and day 42 (*p* < 0.001, Figure 5G). By comparing OFT data differences at the three-time points in the CUMS + FXT group, significant differences were observed in pairwise comparisons at each time point (*p* < 0.001, Figure 5G). Based on the four regions delineated by the L1 and L2 planes of the γ-μ planes, we observed that after administering the FXT positive drug, the CUMS + FXT group shifted from Zones 1 and 4 toward the Control group d that after administering the FXT positive drug, the to determine the mood state of CUMS mice.

## 4 Discussion/conclusion

In recent years, the use of artificial intelligence and mathematical algorithms for detailed behavioral analysis across species, from rodents to primates, has garnered widespread attention (Tseng et al., 2023a; Weinreb et al., 2024). For example, Tseng et al. introduced a method based on a hierarchical three-dimensional motion learning framework (Tseng et al., 2023b). The Hu group investigated the potential of an automated behavioral analysis approach rooted in neural circuits to differentiate depression and aggressive behaviors (Xin and Hu, 2025). Meng et al. applied deep learning techniques to automate the analysis of the FST and tail suspension test TST (Meng et al., 2024). However, processing large datasets requires substantial computational resources and incurs high costs, and as a result, the accuracy of behavioral classification still necessitates manual verification. Additionally, challenges remain in achieving high-throughput simultaneous analysis. Our findings revealed that mice exposed to CUMS, ES and CRS displayed a characteristic LF velocity distribution. Furthermore, it was possible to distinguish mice exhibiting symptoms of anxiety or depression by analyzing the γ and μ parameters derived from LF statistics. This finding offers a novel, illustrative method to differentiate anxiety, depression, and healthy mice. This method is advantageous because it provides a one-step gentle approach (free walk in an OFT) instead of the traditional multi-step stressful tests. This innovative approach has the potential to alleviate the industry’s long-standing concerns regarding the strong stress that traditional tests impose on animals.

While some previous studies have also reported the behavioral features of stressed mice, few statistical models have been developed to help differentiate and quantify highlighted rodent models (Tunc-Ozcan et al., 2019). For instance, Jin et al. reported that mice exhibiting anxiety-like behavior displayed a propensity for increased exploration of the outer area of the box while reducing their time spent in the central region (Fan et al., 2019). Ducottet and Belzung (2005) mentioned that mice unable to escape in a confined space with increased immobility time have depressive-like behavior. These observations illuminate the correlation between rodents’ mental stages and their behaviors. However, when rodents exhibit mild symptoms or when the therapeutic benefits are not readily observable, identifying differences in behavioral markers associated with depression and anxiety can be a challenging task. Additionally, there are variations in the behavioral cues utilized to evaluate models of depression and anxiety (Zhang et al., 2017). Of course, we should also consider that the order of tests may impact the experimental outcomes. To minimize additional stress on rodents, we plan to incorporate the OFT exclusively for assessing depression and anxiety after modeling. Additionally, we will employ the LF method to evaluate the stress levels of rodents. With our new approach, it is possible to process the OFT videos of rodents in an automated batch fashion, enabling the acquisition of quantitative results with minimal stress imposed on the animals.

When utterly ignorant of the orientation and distance of the prey, an animal may unintentionally choose a searching strategy among a few choices of styles. Ralf Metzler et al. calculated that searching trajectories that follow the LF statistics have higher efficiency than those following the normal statistics when the prey is relatively far from the initial starting location (Humphries et al., 2010). On the contrary, if the prey is initially close to the starting position, a normal statistics trajectory is of higher efficiency (Palyulin et al., 2014). Therefore, it is intuitive to assume that evolution has taught many animals a survival strategy such that a decision is made between the normal distribution and the LF distribution based on the expected prey distance. Animals in various stressed mental stages may have different prey distance expectations and thus adopt various searching strategies. Our findings imply that the governing parameters of LF statistics may quantify a mental stage’s severity.

Lack of food is the most common stress animals face in nature. When food and other resources are abundant, mobile organisms tend to forage in a pattern of Brownian motion (Sims et al., 2012). In contrast, when faced with food and other resources shortage pressure, they often present an LF searching strategy, which means the distribution of traveling distances decays as a power law (Barthelemy et al., 2008; Humphries et al., 2010). The stress response of food deficiency in nature is similar to the fear of the unknown for the anxiety and depression mice. The anxiety and depression mice display heavy-tailed distributions in an open field and are consistent with the LF employed by animal predators searching for scarce food sources. In contrast, the speed of non-stress mice performed a normal distribution. A random walk is a nature-inspired algorithm consisting of consecutive unexpected steps or velocities. The LF distribution model usually exists in animal trajectories under survival pressure. This movement trajectory can effectively improve the survival rate when food is scarce. LF has also been applied to other research fields related to bionic (Viswanathan et al., 1999; Palyulin et al., 2014). Although little is known about how LF searching systems developed, evidence indicates that this strategy may have evolved to allow animals to cover the most expansive area with the least energy, which may explain the widespread occurrence of such patterns among modern animals. We suspect that this pattern of behavior, imprinted in animal brains by survival advantage in evolution, should also exist in laboratory animals.

FXT has been well-reported to alleviate the symptoms of CUMS (Dulawa et al., 2004). As anticipated, it was observed that the administration of the drug caused a shift in the mice’s behaviors on the γ–μ plane toward the healthy region from Figures 5D,E (blue dots). On the other hand, the symptoms of the CUMS + saline group appear to be more severe (as indicated by the red dots). Next, we conducted a time-based analysis of the behavioral tests of each mouse in the group identified as the CUMS + saline group. Figure 3F illustrates that their positions on the γ–μ plane shifted gradually from the upper right region (black) to the middle region (pink) and then to the lower left region (yellow) through time, indicative of an increase of severity in their symptoms. Similarly, the behaviors of the CUMS + FXT group were analyzed over time, and it was observed that they initially resided in the upper right region (black) of the γ–μ plane, which then transitioned to the middle region (red), and eventually recovered to the upper right region (yellow). These observations suggest that our quantification method is capable of demonstrating the effects caused by positive drug administration. With further rigorous validations in the future, there is reason to be optimistic that the length of the dot-shifting vector in the γ-μ plane could serve as a quantitative measure of a drug’s therapeutic efficacy.

Chronic stress animal models are extensively utilized in the investigation of the behavioral mechanisms underlying mental disorders. Common models include CUMS, CRS, ES, social isolation stress, and social defeat stress. These models replicate, to varying extents, the mechanisms underlying depression and anxiety-like symptoms in humans (Meng et al., 2024). For example, CUMS is a classic method that induces depressive-like behaviors, such as anhedonia, in animals through repeated and unpredictable mild stressors. While it is highly valid in replicating the core phenotypes of depression, it is highly sensitive to experimental conditions and suffers from poor reproducibility (Markov and Novosadova, 2022; Antoniuk et al., 2019). CRS is commonly used to simulate long-term stress due to its simplicity. The behavioral, neuroendocrine, and brain region functional changes it induces closely resemble those observed in human depression; however, there are significant differences in sensitivity across species and with varying stress intensities (Mao et al., 2022; Tran and Gellner, 2023). Although these stress models have been widely employed in preclinical research, the underlying biological mechanisms of depression and anxiety remain unclear. As a result, the validation of these models primarily depends on behavioral assessments, which are influenced by numerous external factors and often lead to considerable variability in experimental outcomes (Tran and Gellner, 2023). Furthermore, most current studies, in their statistical analyses, include all animals from the same stress model in a unified analysis, overlooking the heterogeneous individual responses to stress, namely the phenotypic differences between stress susceptibility (susceptible) and stress resilience (resilient). Research, such as that involving the chronic social defeat stress model, clearly demonstrates that different individuals exhibit markedly distinct behavioral and neurophysiological responses to the same stress conditions. In these studies, resilient mice exhibit behaviors more similar to the control group, whereas susceptible mice display pronounced depressive or anxiety-like behaviors (Wang et al., 2021; Han et al., 2017).

In this work, we proposed for the first time that LF can distinguish depression and anxiety mice in an OFT. Furthermore, we used speed as a surrogate indicator since stressed animals should subconsciously increase their speed after activating LF search patterns. Finally, we found that the velocity distribution of the stressed mice in the OFT matched that of LF. The r-good value was significantly different from that of the normal mice. At the same time, depression and anxiety groups of mice also showed significant differences in γ and μ parameters, which we could easily distinguish (Edwards, 2008). Based on the velocity LF fitting pattern of the stressed mice in OFT, we could qualitatively evaluate the anxious or depressive state of the tested mice and the efficacy of novel therapeutic agents in a rapid, straightforward, and low-cost way.

In the upcoming discussion, we aim to analyze the significance of the parameters associated with the LF distribution. Given the inherent abstractness of these parameters, they may be challenging to visualize, thereby warranting a need for appropriate visualization methods. To this end, we propose using a visualization technique known as “typical mice” to represent each category and demonstrate the respective groups’ statistical behavior. To construct the typical healthy mouse, we computed the average γ and μ parameters of the control mice group, yielding γ = 1.182 and μ = 0.205, respectively. This typical mouse was named Healthy Doe. Similarly, we derived the Depress Doe (γ = 0.380 and μ = 0.005) and the Anxiety Doe (γ = 0.917 and μ = 0.095) by computing the average values of the corresponding groups. These parameters are plugged into Equation 2 in the Method section and the typical velocity distributions of each group are plotted. In order to plot the typical velocity distributions for each group, the γ and μ parameters obtained for the respective group were inputted into Equation 2, as described in the Method section. The resulting output from this equation provides a graphical representation of the distribution of velocities exhibited by each group. These distributions were then plotted and can be used to compare and contrast the statistical behavior of each group (Supplementary Figure 2). In this article, we used SVM for comparison, and distinguished the control group, depressed and anxious-like model mice, obtaining two dividing lines, L1 and L2. Future studies with larger, multi-center datasets will further refine these regional boundaries to enhance clinical applicability.

A hitherto undiscovered statistical gait pattern in mice exhibiting symptoms of anxiety and depression is unveiled by this investigation. These results introduce a novel and uncomplicated framework for distinguishing between anxiety, depression, and healthy mice. Contrary to conventional and burdensome multi-step tests, this method offers a non-invasive single-step approach (unrestrained ambulation in an open field) that is gentle in nature.

## Data Availability

The data that support the findings of this study are not publicly available due to privacy restrictions but are available from the corresponding author upon reasonable request.
