# Supplementary material for: Lévy statistics define anxiety and depression in mice subjected to chronic stress
Source: Front Behav Neurosci. 2026 Jul 21;20:1757347. doi: 10.3389/fnbeh.2026.1757347 (PMC13433764; doi:10.3389/fnbeh.2026.1757347)
Supplement: Supplementary file 1 [file Data_Sheet_1.zip › Lévy Flight Statistics Software/User Manual for Lévy Flight Statistics Software.pdf]

1. Software Overview

Designed for analyzing and processing mouse activity videos, this software extracts movement speed data, fits the velocity distribution data using a Lévy flight model, and calculates the corresponding fitting parameters.

2. Operation Steps

Main Steps:

Import Video Files

（1）Open the software and click the "Select File" button to choose the video file you want to process. Alternatively, you can drag and drop video files or folders containing videos directly into the video list.

Note: We highly recommend using the .avi format. Please ensure the video quality is clear.

（2）Select Region of Interest (ROI)

In the video display area on the right, select the specific region containing the mouse you wish to track. Please try to match the size of the area as closely as possible to the reference diagram.

（3）Start Processing

Click the "Save" button, then follow the pop-up prompts to begin processing the file.

（4）Export Data Results

Once the processing is complete, the software will display the calculated fitting parameters. Click the "Export Data" button in the bottom left corner, select a destination folder, and save your results.
