# Supplementary material for: Lévy statistics define anxiety and depression in mice subjected to chronic stress
Source: Front Behav Neurosci. 2026 Jul 21;20:1757347. doi: 10.3389/fnbeh.2026.1757347 (PMC13433764; doi:10.3389/fnbeh.2026.1757347)
Supplement: Supplementary file 2 [file Supplementary_file_1.doc]

**Supplementary materials**

Supplementary figure legends

**Figure supplement 1.** The CUMS data was collected both before and after the modeling process.

(**A**) To assess anhedonia in mice, the sucrose preference (SP) was evaluated prior to the experiment on Day -2. Mice with a sucrose preference rate of less than 80% were excluded from the experiment, and a total of 11 mice were eliminated. (**B**) On Day 0, physical evaluations of mice were conducted using the OFT. Mice with fewer than five entries in the center were excluded from the experiment, leading to the elimination of the thirty-one mice. (**C**) To assess anhedonia in mice, the SP was evaluated following CUMS. Mice with a preference rate of less than 80% were excluded from the CUMS group, resulting in the elimination of eight mice. However, the control group only had three mice eliminated. (**D**) On Day 14, the CUMS mice with fewer than five entries in the center in the OFT were excluded, leading to the elimination of seven mice. Regrettably, six mice, comprising two from the control group and four from the CUMS group, succumbed to starvation or mortality during the course of the experiment. Therefore, the final number of animals included 27 in the control group and 52 in the CUMS group. The CUMS mice exhibited a shorter travel distance than the unstressed controls (F_26,51_ = 1.389, *p* < 0.001 for Control vs. CUMS). The presented data represent mean ± s.e.m. Statistical significance was established when p values were below 0.05. The number of animals is indicated on graphs. Two-group comparisons were evaluated with unpaired t-tests.

**Figure supplement 2.** Probabilistic and velocity parameters

Probabilistic and velocity parameters were obtained by averaging miu for each Lévy flight (LF) model and averaging gamma. The resulting averages were then entered into the LF function to generate a set of curves. Three sets of three curves were drawn in this manner.
